# The “Balance of Nature”—Evolution of a Panchreston

**DOI:** 10.1371/journal.pbio.1001963

**Published:** 2014-10-07

**Authors:** Daniel Simberloff

**Affiliations:** Department of Ecology and Evolutionary Biology, University of Tennessee, Knoxville, Tennessee, United States

## Abstract

The earliest concept of a balance of nature in Western thought saw it as being provided by gods but requiring human aid or encouragement for its maintenance. With the rise of Greek natural philosophy, emphasis shifted to traits gods endowed species with at the outset, rather than human actions, as key to maintaining the balance. The dominance of a constantly intervening God in the Middle Ages lessened interest in the inherent features of nature that would contribute to balance, but the Reformation led to renewed focus on such features, particularly traits of species that would maintain all of them but permit none to dominate nature. Darwin conceived of nature in balance, and his emphasis on competition and frequent tales of felicitous species interactions supported the idea of a balance of nature. But Darwin radically changed its underlying basis, from God to natural selection. Wallace was perhaps the first to challenge the very notion of a balance of nature as an undefined entity whose accuracy could not be tested. His skepticism was taken up again in the 20th century, culminating in a widespread rejection of the idea of a balance of nature by academic ecologists, who focus rather on a dynamic, often chaotic nature buffeted by constant disturbances. The balance-of-nature metaphor, however, lives on in large segments of the public, representing a fragile aspect of nature and biodiversity that it is our duty to protect.

## Abstract 2

The notion of a “balance of nature” stretches back at least to the ancient Greeks, but with widely varying conceptions. Originally seen as bestowed by God to protect humankind, after Darwin, the balance came to be seen as fragile, arising by natural selection, and requiring human assistance for maintenance.

The notion of a “balance of nature” stretches back to early Greeks, who believed gods maintained it with the aid of human prayers, sacrifices, and rituals [1]. As Greek philosophers developed the idea of natural laws, human assistance in maintaining the balance did not disappear but was de-emphasized. Herodotus, for instance, the earliest known scholar to seek biological evidence for a balance of nature, asked how the different animal species each maintained their numbers, even though some species ate other species. Amassing facts and factoids, he saw divinely created predators' reproductive rates lower than those of prey, buttressing the idea of a providentially determined balance with a tale of a mutualism between Nile crocodiles beset with leeches and a plover species that feeds on them [1]. Two myths in Plato's *Dialogues* supported the idea of a balance of nature: the Timaeus myth, in which different elements of the universe, including living entities, are parts of a highly integrated “superorganism,” and the Protagoras myth, in which gods created each animal species with characteristics that would allow it to thrive and, having run out of biological traits, had to give man fire and superior intelligence [1]. Among Romans, Cicero followed Herodotus and Plato in advancing a balance of nature generated by different reproductive rates and traits among species, as well as interactions among species [1].

The Middle Ages saw less interest in such pre-set devices as differential reproductive rates to keep nature in balance, perhaps because people believed in a God who would maintain the balance by frequent direct intervention [1]. The Reformation, however, fostered further development of the concept of a providential balance of nature set in motion at creation. Thomas Browne [2] added differential mortality rates to factors maintaining the balance, and Matthew Hale [3] proposed that lower rates of mortality for humans than for other animals maintain human dominance within a balanced nature and added vicissitudes of heat from the sun to the factors keeping any one species from getting out of hand.

The discovery of fossils that could not be ascribed to known living species severely challenged the idea of a God-given balance of nature, as they contradicted the idea of species divinely created with the necessary features for survival [4]. John Ray [5] suggested that the living representatives of such fossils would be found in unexplored parts of the earth, a solution that was viable until the great scientific explorations of the late 18th and early 19th centuries [4]. Ray also argued that what would now be termed different Grinnellian ecological niches demonstrated God's provision of each species with a space of its own in nature.

According to Egerton [1], the earliest use of the term “balance” to refer specifically to ecology was probably by Ray's disciple, William Derham [6], who asserted in 1714 that:

“The Balance of the Animal World is, throughout all Ages, kept even, and by a curious Harmony and just Proportion between the increase of all Animals, and the length of their Lives, the World is through all Ages well, but not over-stored.”

Derham recognized that human populations seemed to be endlessly increasing but saw this fact as a provision by God for future disasters. This explanation contrasts with that of Linnaeus [7], who saw human and other populations endlessly increasing but believed the size of the earth was also increasing to accommodate them. Derham grappled with the issue of theodicy but failed to reconcile plagues of noxious animals with the balance of nature, seeing them rather as “Rods and Scourges to chastise us, as means to excite our Wisdom, Care, and Industry” [1].

Derham's contemporary Richard Bradley [8],[9] focused more on biological facts and less on Providence in sketching a more comprehensive account of an ecological balance of nature, taking account of the rapidly expanding knowledge of biodiversity, noting that each plant had its phytophagous insects, each insect its parasitic wasps or flies and predatory birds, concluding that “all Bodies have some Dependence upon one another; and that every distinct Part of Nature's Works is necessary for the Support of the rest; and that if any one was wanting, all the rest must consequently be out of Order.” Thus, he saw the balance as fragile rather than robust, in spite of a constantly intervening God. Linnaeus [10] similarly marshaled observations of species interactions to explain why no species increases to crowd out all others, adding competition to the predation, parasitism, and herbivory adduced by Bradley and also emphasizing the different roles (we might now say “niches”) of different species as allowing them all to coexist in a sort of superorganismic, balanced whole.

Unlike Derham, Georges-Louis Leclerc, Comte de Buffon [11] managed to reconcile animal plagues with a balanced nature. He perceived the balance of nature as dynamic, with all species fluctuating between relative rarity and abundance, so that whenever a species became overabundant, weather, predation, and competition for food would bring it back into balance. Buffon's successor as director of the Jardin des Plantes in Paris, Jacques-Henri Bernardin de Saint-Pierre [12], was probably the first to associate ecological damage caused by biological invasions with a disruption of the balance of nature. Observing damage to introduced trees from insects accidentally introduced with them, he argued that failure to introduce the birds that would eat the insects led to the damage. William Paley [13], perhaps the inspiration for today's advocates of “intelligent design,” analogized nature to a watch. One would assume a smoothly running watch was designed with purpose, and so too nature was designed by God with balance and a purpose.

In the 19th century, evolution burst on the scene, greatly influencing and ultimately modifying conceptions of a balance of nature. Fossils that seemed unrelated to any living species, as noted above, conflicted with the balance of nature, because they implied extinction, a manifestly unbalanced event that furthermore could be seen to imply that God had made a mistake. Whereas Ray had been able to argue that living exemplars of fossil species would be found in unexplored parts of the earth, by the 19th century, this explanation could be rejected. Jean-Baptiste Lamarck [14] resolved the conflict in a different way, arguing that species continually change, so the balance remains the same. The fossils thus represent ancestors of living species, not extinct lineages. Robert Chambers [15], another early evolutionist, similarly saw fossils not as a paradox in a balanced nature but as a consequence of the fact that, as the physical environment changed, species either evolved or went extinct.

Alfred Russel Wallace was perhaps the first to question the very existence of a balance of nature, in a remarkable notebook entry, ca. 1855:

“Some species exclude all others in particular tracts. Where is the balance? When the locust devastates vast regions and causes the death of animals and man, what is the meaning of saying the balance is preserved… To human apprehension there is no balance but a struggle in which one often exterminates another” [16].

In modern parlance, Wallace appears almost to be asking how “balance” could be defined in such a way that a balance of nature could be a testable hypothesis.

Darwin's theory of evolution by natural selection certainly explained the existence of fossils, and his emphasis on inevitable competition both between and within species downplayed the role of niche specialization propounded by Plato, Cicero, Linnaeus, Derham, and others [1]. Darwin nevertheless saw the ecological roles of the diversity of species as parts of an almost superorganismic nature, and his main contribution to the idea of a balance of nature was his constant emphasis on competition and other mortality factors that kept all species' populations in check [1]. His many metaphors and examples of the interactions among species, such as the tangled bank and the spinsters-cats-mice-bumblebees-clover stories in *The Origin of Species* [17], contributed to a sense of a highly balanced nature, but one driven by natural selection constantly changing species, rather than by God either intervening or creating species with traits that ensure their continued existence. Unlike Wallace, Darwin did not raise the issue of whether nature was actually balanced and how we would know if it was not.

As ecology developed in the late 19th and early 20th centuries, it was inevitable that Wallace's question—how to define “balance”—would be raised again and that increasingly wide and quantitative study, especially at the population level, would be brought to bear on the matter. The work of the early dominant plant ecologist Frederic Clements and his followers, with Clements' notion of superorganismic communities [18], provided at least tacit support for the idea of a balance of nature, but his contemporary Charles Elton [19], a founder of the field of animal ecology and a leading student of animal population cycles, forcefully reprised Wallace's concern:

“‘The balance of nature’ does not exist, and perhaps never has existed. The numbers of wild animals are constantly varying to a greater or lesser extent, and the variations are usually irregular in period and always irregular in amplitude. Each variation in the numbers of one species causes direct and indirect repercussions on the numbers of the others, and since many of the latter are themselves independently varying in numbers, the resultant confusion is remarkable.”

Despite Elton's explicit skepticism, his depiction of energy flow through food chains and food webs was incorporated as a superorganismic analog to the physiology of individuals (e.g., [20]). Henry Gleason, another critic of the superorganism concept, who depicted populations distributed independently, rather than in highly organized communities, was ignored at this time [21].

However, beginning with three papers in *Ecological Monographs* in 1947, the superorganism concept was increasingly questioned and, within 25 years, Gleason was vindicated and his views largely accepted by ecologists [22]. During this same period, extensive work by population biologists again took up Elton's focus on population trajectories and contributed greatly to a growing recognition of the dynamism of nature and the fact that much of this dynamism did not seem regular or balanced [21]. The idea of a balanced nature did not immediately disappear among ecologists. For instance, a noteworthy book by C. B. Williams [23], *Patterns in the Balance of Nature*, described the distribution of abundances within communities or regions as evincing statistical regularity that might be construed as a type of “balance of nature,” at least if changes in individual populations do not change certain statistical features (a hypothesis that Williams considered untested at the time). But the predominant view by ecologists of the 1960s saw the whole notion of a balance as, at best, irrelevant and, at worst, a distraction. Ehrlich and Birch [24], for example, ridiculed the idea:

“The existence of supposed balance of nature is usually argued somewhat as follows. Species X has been in existence for thousands or perhaps millions of generations, and yet its numbers have never increased to infinity or decreased to zero. The same is true of the millions of other species still extant. During the next 100 years, the numbers of all these species will fluctuate; yet none will increase indefinitely, and only a few will become extinct… Such ‘observations’ are made the basis for the statement that population size is ‘controlled’ or ‘regulated,’ and that drastic changes in size are the results of upsetting the ‘balance of nature.’”

Another line of ecological research that became popular at the end of the 20th century was to equate “balance of nature” with some sort of equilibrium of numbers, usually of population sizes [25], but sometimes of species richness. The problem remained that, with numbers that vary for whatever reason, it is still arbitrary just how much temporal variation can be accommodated within a process or phenomenon for it still to be termed equilibrial [26]. Often the decision on whether to perceive an ecological process as equilibrial seems to be based on whether there is some sort of homeostatic regulation of the numbers, such as density-dependence, which A. J. Nicholson [27] suggested as an argument against Elton's skepticism of the existence of a balance. The classic 1949 ecology text by Allee et al. [28] explicitly equated balance with equilibrium and cited various mechanisms, such as density-dependence, in support of its universality in nature [25]. Later similar sorts of mathematical arguments equated the mathematical stability of models representing nature with a balance of nature [29], although the increasing recognition of stochastic aspects and chaotic mathematics of population fluctuations made it more difficult to perceive a balanced nature in population trajectories [21].

For academic ecologists, the notion of a balance of nature has become passé, and the term is widely recognized as a panchreston [30]—a term that means so many different things to different people that it is useless as a theoretical framework or explanatory device. Much recent research has been devoted to emphasizing the dynamic aspects of nature and prominence of natural or anthropogenic disturbances, particularly as evidenced by vicissitudes of population sizes, and advances the idea that there is no such thing as a long-term equilibrium (e.g., [31],[32]). Some authors explicitly relate this research to a rejection of the concept of a balance of nature (e.g., [33]–[35]), Pickett et al. [33] going so far as to say it must be replaced by a different metaphor, the “flux of nature.”

The issue is confounded by the fact that the perception of balance can be sought at different levels (populations, communities, ecosystems) and spatial scales. Much of the earlier discussion of a balance was at the population and community levels—Browne, Hale, Bradley, Linnaeus, Buffon, Bernardin de Saint-Pierre, and Darwin saw balance in the limited fluctuations of populations and the interactions of populations as one force imposing the limits. The proponents of density-dependent population regulation fall in this category as well [36],[37]. As a balance is sought at the community and ecosystem levels, the sorts of evidence brought to bear on the matter become more complicated and abstract [37],[38]. It is increasingly difficult to imagine what sorts of empirical or observational data could test the notion of a balance. For instance, Williams's balance of nature—evidenced by a particular statistical distribution of population sizes—would not be perceived as balanced by many observers in light of the fact that entire populations can crash, explode, or even go extinct within the constraint of a statistical distribution of a given shape. Early claims of a balance at the highest level, such as the various superorganisms (Plato's Timaeus myth, Paley's watch metaphor, Clements's superorganismic plant community) can hardly be seen as anything other than metaphors rather than testable hypotheses and have fallen from favor. The most expansive conception of a balance of nature—the Gaia hypothesis [39]—has been almost universally rejected by scientists [40]. The advent and growing acceptance of the metapopulation concept of nature [41] also complicates the search for balance in bounded population fluctuations. Spatially limited individual populations can arise, fluctuate wildly, and even go extinct, while suitable dynamics maintain the widespread metapopulation as a whole.

Yet, the idea of a balance of nature lives on in the popular imagination, especially among conservationists and environmentalists. However, the usual use of the metaphor in an environmental context suggests that the balance, whether given by God or produced by evolution, is a fragile balance, one that needs human actions for its maintenance. Through the 18th century, the balance of nature was probably primarily a comforting construct—it would protect us; it represented some sort of benign governance in the face of occasional awful events. When Darwin replaced God as the determinant of the balance with natural selection, the comfort of a balance of nature was not so overarching, if there was any comfort at all. Today, ecologists do not even recognize a balance, and those members of the public who do, see it as something we must protect if we are ever to reap benefits from it in the future (e.g., wetlands that might help ameliorate flooding from storms and sea-level rise). This shift is clear in the writings of Bill McKibben [42],[43], who talks frequently about balance, but about balance **with** nature, not balance **of** nature, and how humankind is headed towards a catastrophic future if it does not act promptly and radically to rebalance society with nature.
